# Tophöse Gicht als Differenzialdiagnose eines präaurikulären Tumors

**DOI:** 10.1007/s00106-022-01253-y

**Published:** 2022-11-30

**Authors:** Amelie Birk, Klaus Wörtler, Carolin Mogler, Katharina Storck

**Affiliations:** 1 Klinik und Poliklinik für Hals‑, Nasen‑, Ohrenheilkunde des Klinikums rechts der Isar, TU München, Ismaninger Str. 22, 81675 München, Deutschland; 2 Sektion Muskuloskelettale Radiologie, Klinikum rechts der Isar, TU München, München, Deutschland; 3 Institut für allgemeine Pathologie Klinikum rechts der Isar, TU München, München, Deutschland

**Keywords:** Arthropathie, Temporomandibulargelenk, Gichttophus, Präaurikuläre Schwellung, Progrediente Hörminderung, Arthropathy, Temporomandibular joint, Gout tophi, Preauricular tumor, Progressive hearing impairment

## Abstract

Die primäre Gicht ist eine erbliche Störung des Nukleotidstoffwechsels. Neben typischen Manifestationen an Füßen, Händen und den großen Gelenken kann es selten zu atypischen Verläufen im HNO-Bereich kommen. Wir berichten von einem Fall der tophösen Gicht im Temporomandibulargelenk mit Symptomen einer präaurikulären Schwellung und progredienter Hörminderung. Klinisch zeigte sich ein verlegter Gehörgang, bildgebend ein destruierender Prozess, der unter anderem die Schädelbasis involvierte. Die Diagnosesicherung gelang radiologisch und bioptisch.

## Abstract (en)

Primary gout is a hereditary disorder in nucleotide metabolism. In addition to typical manifestations in the feet, hands, and large joints, there may be rare manifestations in the head and neck. We report a case of tophaceous gout in the temporomandibular joint in a patient who presented with preauricular swelling and progressive hearing impairment. Physical examination showed obliteration of the auditory canal and imaging revealed a destructive process involving the skull base. The diagnosis was confirmed by imaging and biopsy.

## Hintergrund

Bei der primären Gicht handelt es sich um eine erbliche bedingte Störung des Nukleotidstoffwechsels, die vor allem als Wohlstandskrankheit auftritt und bevorzugt Männer jenseits des 40. Lebensjahres betrifft [1]. Bei hohen Harnsäurespiegeln kommt es zu Ablagerungen von Uratkristallen v. a. in den Gelenken, die als akute Gicht in Form einer typischen schmerzhaften Monoarthropathie, z. B. am Großzehen- oder Daumengrundgelenk, oder als chronische Form polyartikulär auftreten können. Arthritische Tophi sind im HNO-Fachbereich am häufigsten an der Ohrmuschel zu finden, Lokalisationen wie das Temporomandibulargelenk sind rar [2–5]. Die Diagnostik umfasst klinische Kriterien und die Gelenkpunktion sowie bildgebende Verfahren wie konventionelle Röntgenaufnahmen, Arthrosonographie und Dual-Energy-Computertomographie (DECT) [6]. In der Akuttherapie kommen vor allem nichtsteroidale Antirheumatika (NSAR) zur Anwendung. In der langfristigen Therapie der Hyperurikämie werden Urikostatika oder Urikosurika eingesetzt. In Einzelfällen, insbesondere bei schwerer tophöser Gicht, kann zusätzliche eine operative Therapie nötig sein. Die medikamentöse Therapie wird in der Regel durch eine purinfreie bzw. -arme Diät begleitet.

## Anamnese

In der Klinik und Poliklinik der Hals‑, Nasen- Ohrenheilkunde des Klinikums rechts der Isar der TU München stellte sich eine 68-jährige Patientin mit schmerzhafter Schwellung präaurikulär linksseitig sowie einer progredienten Hörminderung seit 6 Monaten ipsilateral vor. Bei der Patientin wurde 3 Jahre zuvor aufgrund derselben Beschwerdesymptomatik die Verdachtsdiagnose einer chronischen Kiefergelenkarthropathie gestellt. Eine damals empfohlene Arthroskopie wurde von der Patientin abgelehnt. Weitere Beschwerden oder chronische Erkrankungen bestanden nicht.

## Befund

Ohrmikroskopisch zeigte sich eine harte, von reizloser Haut überzogene Verlegung des linken Gehörgangs. Außerdem bestand eine leicht druckdolente Schwellung präaurikulär ohne Kieferklemme. Die weitere Spiegeluntersuchung, sowie die übrige klinische Untersuchung waren unauffällig. Serologisch zeigte sich ein erhöhter Harnsäurewert von 6,1 mg/dl (Normbereich: 2,5–5,5 mg/dl). Die Entzündungsmarker und Nierenretentionsparameter waren unauffällig. Audiometrisch ergab sich eine Schallleitungsschwerhörigkeit linksseitig bei Normakusis rechtsseitig.

**Figure 1:**
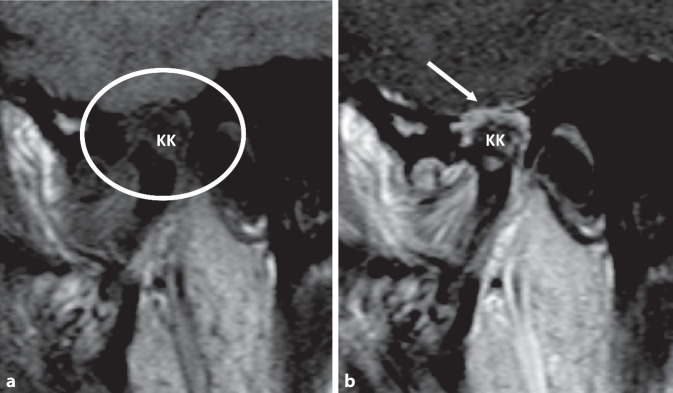

In einer bereits durchgeführten 3 Jahre alten Magnetresonanztomographie (MRT) des Temporomandibulargelenks (Abb. 1) stellte sich ein destruktiver Prozess des linken Kiefergelenks mit Beteiligung des knöchernen Caput mandibulae und der Gelenkpfanne dar, ebenso wie Anzeichen einer proliferativen Synovialitis.

**Figure 2:**
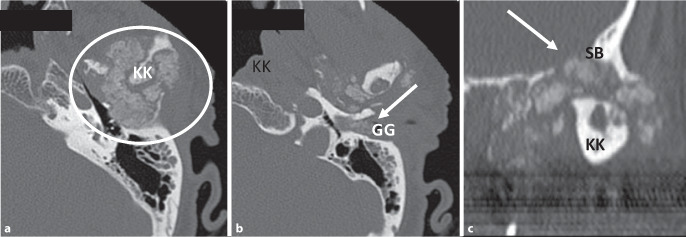

Die nachfolgend durchgeführte CT des Felsenbeins (Abb. 2) zeigte einen kalzifizierenden Prozess, ausgehend vom linken Kiefergelenk. Dieser den Knochen arrosierende Prozess involvierte bildmorphologisch Caput mandibulae, Fossa mandibularis und die mittlere Schädelgrube mit expansiv vorwachsenden Kalzifikationen gegen das Mittelohr und den äußeren Gehörgang. Pathologische Lymphknoten konnten nicht detektiert werden.

**Figure 3:**
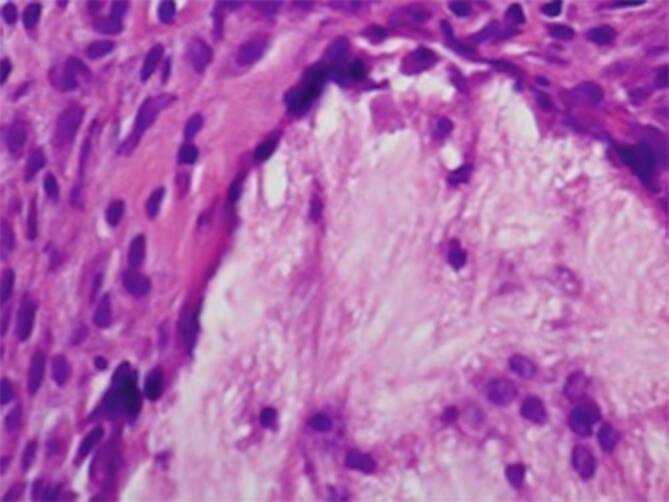

Es folgte eine Biopsie, deren histologische Aufarbeitung landkartenartig verzweigte, büschelartige kristalline stromale Einlagerungen umgeben von einem fibrovaskulären Weichgewebswall mit mehrkernigen Riesenzellen zeigten, passend zu einem Gichttophus (Abb. 3).

## Therapie und weiterer Verlauf

Nach Diagnosesicherung des Gichttophus im Temporomandibulargelenk, wurden weitere Gichtmanifestationen an anderer Stelle klinisch ausgeschlossen. Aufgrund der geringen Beschwerdesymptomatik und der erheblichen Ausdehnung des Befundes fiel die Entscheidung gegen ein operatives Verfahren.

Für die Akuttherapie erhielt die Patientin NSAR. Zusätzlich wurde eine Therapieeinleitung mit Xanthinoxidasehemmern bei Hyperurikämie und die Einhaltung einer purinarmen Diät empfohlen. Eine erneute Kontrolle mittels CT erfolgte 2 Jahre nach Erstvorstellung, dabei zeigte sich keine Größenprogredienz. Die Patientin war zu diesem Zeitpunkt schmerzfrei.

## Diskussion

Die primäre Gicht entsteht durch ein Ungleichgewicht zwischen der tubulär renalen Ausscheidung von Harnsäure und einer erhöhten Produktion bei erhöhter Purinaufnahme. Es handelt sich um eine genetische Erkrankung, die durch Fehlernährung verstärkt wird. Bei der viel selteneren sekundären Gicht handelt es sich dagegen um eine Hyperurikämie infolge eines allgemein erhöhten Nukleotidumsatzes im Rahmen einer anderen Grunderkrankung, z. B. bei onkologischen Erkrankungen. Mononatriumuratkristalle lagern sich besonders bei niedrigen Temperaturen in der Synovialflüssigkeit an, daher gehäuft an den Extremitäten. Der Verlauf in Stadien reicht von einer asymptomatischen Hyperurikämie über die akute und interkritische Gicht bis zur chronischen/tophösen Gicht [1]. Die Beteiligung des Kiefergelenks im Rahmen einer Gichterkrankung ist äußerst selten und wurde bisher nur in einzelnen Fallberichten beschrieben. Andere seltene Manifestationen im HNO-Bereich wurden an Nasenlöchern, Zunge, Epiglottis, Stimmband, Arytänoidknorpel oder Schildknorpel mit entsprechenden klinischen Befunden und Funktionseinschränkungen beschrieben [7–10]. In den zitierten Fallberichten präsentierte sich der Kiefergelenkbefall ähnlich wie im dargestellten Kasus gehäuft mit einer progredienten Hörminderung aufgrund einer Gehörgangverlegung, Tragusschmerzen [2], präaurikulären Schwellungen [4], Schmerzen im Bereich des Kiefergelenks [2, 3, 5] sowie mit einer eingeschränkten Mundöffnung [5]. Die üblichen bildgebenden Verfahren (OPG, CT, MRT) reichten in diesen Fällen nicht aus, um eine sichere Diagnose zu stellen. In konventionellen Röntgenaufnahmen werden tophöse Gichtmanifestationen oft als relativ dichte Weichteilprozesse mit ossären Erosionen, Gelenkdestruktionen, überhängenden Knochenrändern („Gichtstachel“) und subchondraler Zystenbildung beschrieben [11], die chronischen Entzündungen oder mitunter Malignomen ähneln können. Mit der Dual-Energy-CT (DECT) können Uratkristalle spezifisch nachgewiesen werden. Die simultane Anwendung zweier unterschiedliche Röntgenenergien ermöglicht die Unterscheidung von Natriumurat und kalziumhaltigen Strukturen aufgrund unterschiedlichen Röntgenabsorptionsverhaltens. Die MRT kann zusätzlich Veränderungen wie eine chronisch proliferative Synovialitis zeigen [11]. Eine Biopsie ist bei Manifestation in atypischen Lokalisationen Mittel der Wahl zur Diagnosesicherung. Therapeutisch wird die Gicht vorrangig medikamentös behandelt und um eine purinarme Diät ergänzt. In Einzelfällen kann bei komplizierter Gicht mit Gelenkdestruktion ein chirurgisches Vorgehen indiziert sein. Aufgrund der Ausdehnung des vorliegenden Befundes mit Schädelbasisbezug wäre operativ mit einem erheblichen Defekt zu rechnen gewesen und wurde von der Patientin abgelehnt. In anderen Fallberichten erfolgte häufig eine chirurgische Exploration mit Resektion über einen präaurikulären Zugang [2, 4], teilweise unter Mitnahme des Unterkieferkondylus bzw. des Meniskus [5]. Postoperativ gelang in allen Fällen, die Schmerzfreiheit der Patienten und die Wiederherstellung des jeweiligen funktionellen Defizits zu erreichen. Im dargestellten Fall hätte eine partielle Resektion des Tophus eine Hörverbesserung erzielen können, wurde jedoch von der Patientin abgelehnt.

## Fazit für die Praxis

- Trotz der steigenden Prävalenz der Gicht ist die Beteiligung des Temporomandibulargelenks eine seltene, aber relevante Differenzialdiagnose einer präaurikulären Schwellung mit gelenkzerstörendem Potenzial.
- In der Literatur finden sich nur vereinzelte Fallberichte.
- Die Therapie erfolgt primär medikamentös.
- In Einzelfällen kann bei komplizierter Gicht ein operatives Vorgehen sinnvoll sein.
