# Erratum zu: Tophöse Gicht als Differenzialdiagnose eines präaurikulären Tumors

**DOI:** 10.1007/s00106-022-01269-4

**Published:** 2023-01-17

**Authors:** Amelie Birk, Klaus Wörtler, Carolin Mogler, Katharina Storck

**Affiliations:** 1 Klinik und Poliklinik für Hals‑, Nasen‑, Ohrenheilkunde, Klinikum rechts der Isar, TU München, Ismaninger Str. 22, 81675 München, Deutschland; 2 Sektion Muskuloskelettale Radiologie, Klinikum rechts der Isar, TU München, München, Deutschland; 3 Institut für allgemeine Pathologie, Klinikum rechts der Isar, TU München, München, Deutschland

**Erratum zu:**

**HNO 2022**

10.1007/s00106-022-01253-y

Die Darstellung von Abb. 2 in diesem Artikel war fehlerhaft. Die Abbildung hätte wie oben aussehen müssen.

**Figure 1:**
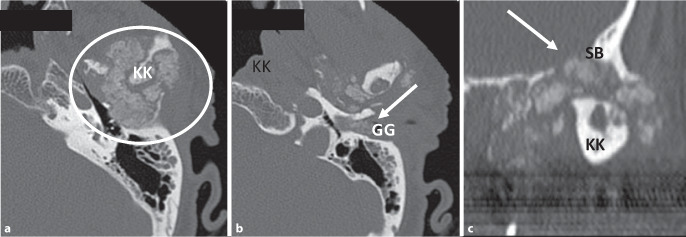

Der Originalbeitrag wurde korrigiert.

